# 4265 Effect of individual characteristics, healthcare access, and built environment on care coordination outcomes related to cardiovascular disease risk factors

**DOI:** 10.1017/cts.2020.356

**Published:** 2020-07-29

**Authors:** Sonal J. Patil, Yan Wang, Angela Johnson, David Mehr, Randi Foraker, Robin Kruse

**Affiliations:** 1 Washington University in St. Louis, Institute Of Clinical and Translational Sciences

## Abstract

OBJECTIVES/GOALS: We examined how individual characteristics and characteristics of the socioeconomic and built environment were associated with care coordination’s effect on cardiovascular disease (CVD) risks to identify geographic areas that may benefit from supplementary clinic-community linkages. METHODS/STUDY POPULATION: We analyzed data with geocoded residential addresses and data from electronic health records for 9946 adults from a Centers for Medicare & Medicaid Services funded innovation project from 7/1/2013 to 3/30/2015. Variables included patient-level demographics, Elixhauser comorbidity index, total time with a nurse care manager, and neighborhood factors such as poverty indicators, walkability, and social capital index. Outcomes were change in CVD risk factors, hemoglobin A1C, blood pressure (BP), and low-density lipoprotein (LDL). Generalized linear models were used to assess the effect of nurse care management program on outcomes after controlling for confounding factors. RESULTS/ANTICIPATED RESULTS: We report preliminary models that include patient demographics (age, sex, race), health care utilization, nurse care manager contact time, Elixhauser comorbidity index, neighborhood education status, percent of population below 200% federal poverty level, median home value, walkability score of the residential address, and social capital index. After adjusting for all mentioned variables, in adults with HbA1C more than 7.5% at baseline, females had worsening HbA1C by 0.53% over the study period. Additionally, LDL values in females worsened over the study period by 4.8 mg/dL after adjusting for all variables. No clinically significant changes were noted for BP. DISCUSSION/SIGNIFICANCE OF IMPACT: Women’s HbA1C and LDL worsened despite nurse care management and may benefit from additional community-based interventions or interventionists. In future analyses, we anticipate that CVD risk will worsen for patients with higher fast food proximity and with greater geographic distance from their PCP.

